# Household Food Insecurity along an Agro-Ecological Gradient Influences Children’s Nutritional Status in South Africa

**DOI:** 10.3389/fnut.2017.00072

**Published:** 2018-01-22

**Authors:** Gamuchirai Chakona, Charlie M. Shackleton

**Affiliations:** ^1^Department of Environmental Science, Rhodes University, Grahamstown, South Africa

**Keywords:** food insecurity, agro-ecological gradient, rural–urban continuum, malnutrition, dietary diversity, stunting, wasting, wild foods

## Abstract

The burden of food insecurity and malnutrition is a severe problem experienced by many poor households and children under the age of five are at high risk. The objective of the study was to examine household food insecurity, dietary diversity, and child nutritional status in relation to local context which influences access to and ability to grow food in South Africa and explore the links and associations between these and household socio-economic status. Using a 48-h dietary recall method, we interviewed 554 women from randomly selected households along a rural–urban continuum in three towns situated along an agro-ecological gradient. The Household Dietary Diversity Scores (HDDS) and the Household Food Insecurity Access Scale (HFIAS) tools were used to measure household dietary diversity and food insecurity, respectively. Anthropometric measurements with 216 children (2–5 years) from the sampled households were conducted using height-for-age and mid-upper arm circumference (MUAC) as indicators of stunting and wasting, respectively. The key findings were that mean HDDS declined with decreasing agro-ecological potential from the wettest site (8.44 ± 1.72) to the other two drier sites (7.83 ± 1.59 and 7.76 ± 1.63). The mean HFIAS followed the opposite trend. Stunted growth was the dominant form of malnutrition detected in 35% of children and 18% of children were wasted. Child wasting was greatest at the site with lowest agro-ecological potential. Children from households with low HDDS had large MUAC which showed an inverse association among HDDS and obesity. Areas with agro-ecological potential had lower prevalence of food insecurity and wasting in children. Agro-ecological potential has significant influence on children’s nutritional status, which is also related to household food security and socio-economic status. Dependence on food purchasing and any limitations in households’ income, access to land and food, can result in different forms of malnutrition in children. Responses to address malnutrition in South Africa need to be prioritized and move beyond relying on food security and nutritional-specific interventions, but rather on nutrition-specific and sensitive programs and approaches; and building an enabling environment. Land availability, agriculture (including climate-smart agriculture especially in drier areas), and wild foods usage should be promoted.

## Introduction

Nutritional status is considered a key indicator of national development, because a well-nourished and healthy population is essential for successful economic and social development. Food insecurity and malnutrition remain a global challenge, especially in sub-Saharan Africa, where the number of the hungry and undernourished people are increasing (1). Many low-income households consume monotonous diets which are of low quality, cereal based, and lack diversity, thereby increasing the risk of micronutrient deficiencies which is already high in these resource-poor settings (2, 3). It is particularly important to have better understanding of the effects of food insecurity on vulnerable groups in societies, especially the poor, women of reproductive age, and children under the age of five as they are at high risk (4–7). Household food insecurity is one determinant of nutritional status of children especially in developing countries (8, 9) as it directly affects the quantity and quality of dietary intake (5, 6, 10). For example, food-insecure households in Columbia and Pakistan had greater probability of having stunted (11, 12) and underweight child (12).

Although previous reports suggested an improved global nutrition situation, a lot still needs to be achieved with an estimated 178 million children younger than 5 years stunted and 55 million wasted, most of which live in sub-Saharan Africa and south-central Asia (13). Child undernutrition remain prevalent in developing countries (7) which is linked to poor quality diets, typically low in calories and essential nutrients (5, 14), although maternal and caregiver education have also been highlighted. About 45% of child deaths have underlying nutritional causes due to suboptimal breastfeeding, poor feeding, care, and aggravated by illness (7, 15). Thus, a major public health problem in developing countries as millions of households live in poverty and have difficulties in accessing nutritious food (9). Poor people tend to adopt unvaried diets mainly of starchy staples such as maize meal, with low vegetable and fruit intake, to cope with poverty (2, 16, 17). The choice and consumption of diverse foods may be limited to higher income households (18) as it is determined by the market price (19, 20) and education level (21, 22). For example, Torlesse et al. (23) reported in Bangladesh on the association between rice prices, rice expenditures, and child undernutrition. The prevalence of underweight children decreased with a decline in rice prices as households were able to spend more money on other nutrient-rich foods (23).

In South Africa, almost 14.3 million South Africans are defined as vulnerable to hunger and 43% of the households are vulnerable to poverty (24, 25), with about 20% living in extreme poverty. The majority of the households cannot afford to purchase sufficient food to provide them with an adequate diet, therefore households which are living below the food poverty line consume poor quality diets and alter their consumption routines to fit with their poverty (26). The greater proportion of the population (especially the urban poor) tend to consume energy rich and processed foods, including refined grains, and foods higher in saturated fat, sugar, and salt which can lead to individuals being overweight (27–29). Due to nutritional transition as many households are moving from rural to urban lifestyles, undernutrition and overnutrition now coexist in the country, affecting the majority of the population (28, 30, 31). The country is among the top 20 countries internationally with the highest burden of undernutrition (32–34), and is listed by the World Health Organisation (WHO) as one of 36 high-burden countries (28). Stunted growth and underweight are the major nutritional disorders affecting the nation (25) although school feeding programs and child support grants have reduced child undernutrition in the country (31, 35). Stunted growth is the dominant form of malnutrition among South African children with about 21–48% of young children suffering from chronic malnutrition (22, 36–38). About 4.5% of children in South Africa were reported to have been suffering from wasting in 2005 (39) and 6.8% (4.8% wasted and 2% severely wasted) in 2008 (40). Faber and Wenhold (41) reported that chronic malnutrition is a bigger problem than acute malnutrition in South Africa, as was also reported in other developing countries like Ghana (9) and Ethiopia (42). Poor access to nutrient-rich foods and availability restricts South African children from consuming adequate and diversified diets with fruits and vegetables, and this has been associated with poor growth and increased levels of stunting and underweight in South African children (16, 43). Household food insecurity is widespread in South Africa, yet there is limited information on its contribution to child malnutrition, especially in different agro-ecological zones (AEZs). No study has explored the links between household food insecurity, household dietary diversity and the nutritional status of children in South Africa, particularly in mid-sized towns, yet these areas are characterized by higher poverty levels which is the major factor influencing food insecurity by limiting households’ food access (44). The study considered AEZs, which are geographical areas exhibiting similar climatic conditions that determine their ability to support rain-fed agriculture as influenced by latitude, elevation, and temperature, as well as seasonality, rainfall amount, and distribution during the growing season (45). These may have influence on household diets and food access. The influence of the rural–urban gradient was examined to fully understand the nature and processes occurring along the gradient. Iaquinta and Drescher (46) argued that the relationship between food production and food insecurity should be evaluated across the entire rural, peri-urban, and urban system, because neither are no longer solely the domain of rural areas as some urban dwellers are also involved in activities that used to be considered for rural habitants only. The study measured and explored the links between household food insecurity, dietary diversity, and child nutritional status in relation to local context which influences access to and ability to grow food. We hypothesized that:

1. Households in the high AEZ would be more food secure than those with lower AEZ because of better climatic conditions that promote farming and lower dependence on food purchasing.
2. Food insecurity would be high in peri-urban locations than in the rural and urban as high levels of poverty, unemployment, and lack of access/entitlements to land makes them more sensitive to changes in income and food prices.
3. Poor households would cope with poverty by reducing the quality, quantity, and number of meals consumed per day.
4. Household dietary diversity would have a significant negative association with household food insecurity.
5. Children from food-insecure households would more likely be stunted or wasted than their counterparts from food secure households.

## Materials and Methods

### Study Area and Sampling

**Figure 1:**
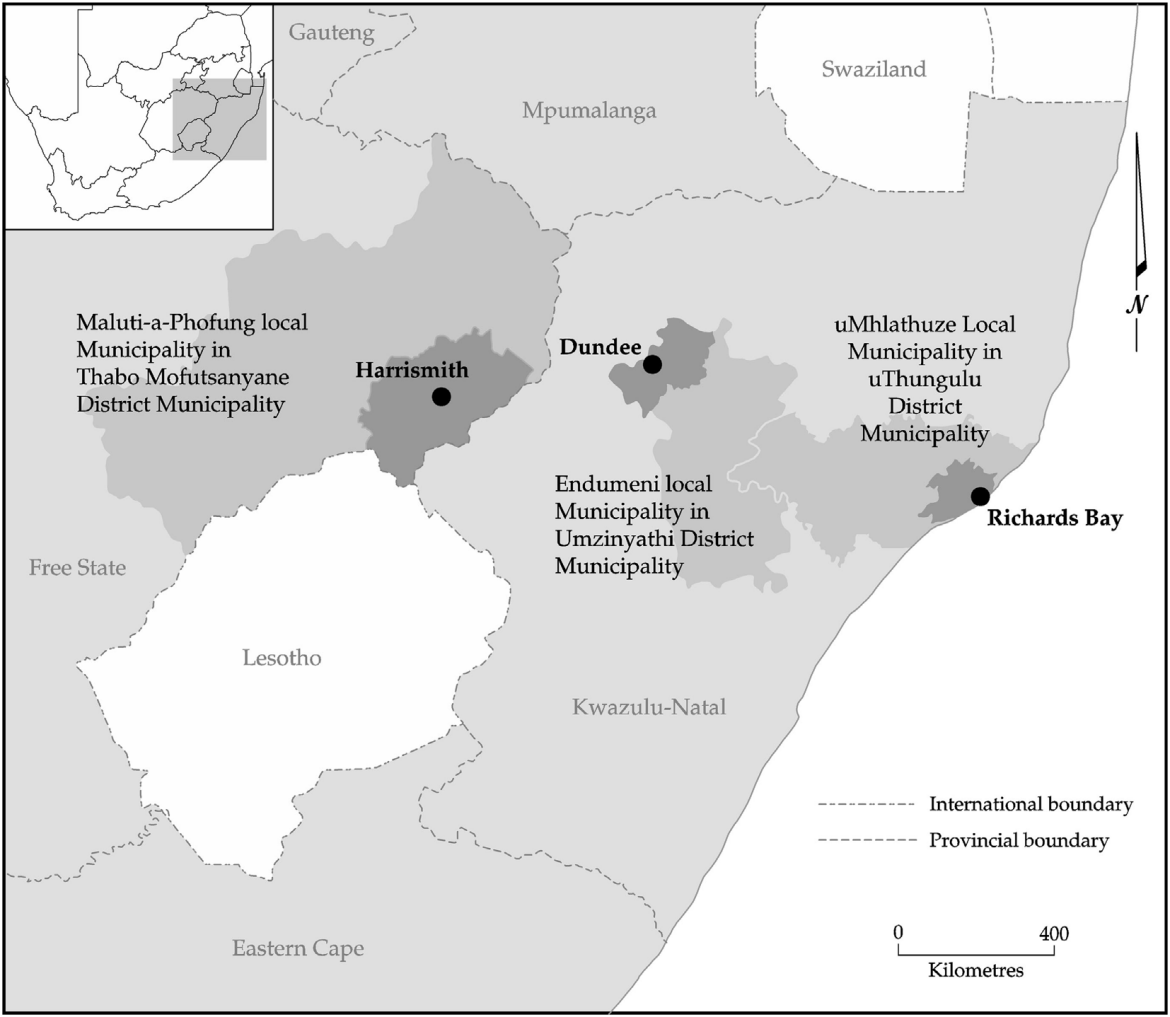
Location of study sites [adopted from Ref. (47)].

Three medium-sized South African towns (Richards Bay, Dundee, and Harrismith) located along an agro-ecological gradient which reflects one of declining suitability for rain-fed agriculture (Figure 1).

**Figure 2:**
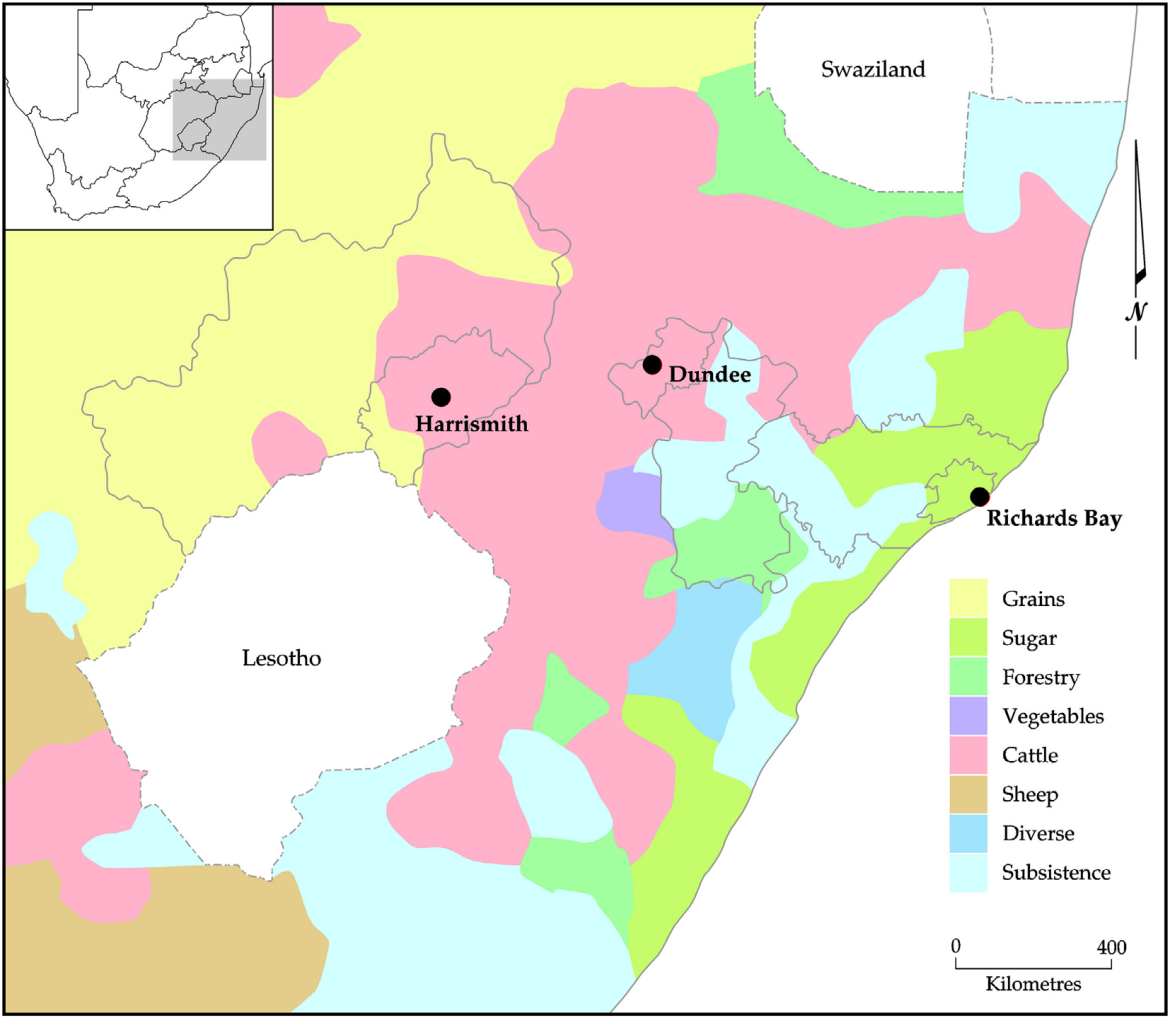
South Africa’s agricultural regions.

The towns were selected according to their position along an agro-ecological gradient, with Richards Bay being a coastal and relatively warm and wet town (approximately 970 mm per annum), while Harrismith is an inland and dry town (approximately 622 mm per annum), and Dundee being intermediate (inland and 683 mm per annum). The seasonality of the rainfall increases along this gradient, along with the severity of winter temperatures (47). Thus, the suitability for rain-fed agriculture declines from high in Richards Bay where there is also longer growing seasons to low in Harrismith, where rural farms mostly practice cattle ranching (Figure 2).

Each study site consisted of the rural, peri-urban, and urban complex and in each town, data were collected along the rural–urban continuum. We used ArcGIS software to randomly select households at each site and GPS coordinates were generated for 200 households in each town, comprising of 60 rural, 80 peri-urban, and 60 urban households. Sampling size in the locations was adopted to limit the time and costs of contacting widely scattered households in rural areas and more households were sampled in the peri-urban locations because many people are migrating to these peri-urban areas in South Africa. Data were obtained through administering questionnaires to randomly selected households at each site. Only a total of 183 individuals in Richards Bay; 173 in Dundee; and 198 in Harrismith agreed to participate in the interviews. Interviews were conducted twice with the same individual [pre-harvest period (October and November 2014) and post-harvest period (June 2015)] to cover for possible variations in seasonal local diets, nutrition, and food access. All interviews were conducted with a woman of reproductive age (15–49 years old) in her preferred language of isiZulu in Richards Bay and Dundee, and Sesotho in Harrismith or English. The person in the household who prepares most of the meals was interviewed. If the person who does most of the cooking was a stay out employee (domestic worker), she was required to report on the meals s/he had cooked for the household and not what she consumed herself. In the case of one the selected households refusing to participate or no women of reproductive age being available for interview, then the nearest house to the left was interviewed. Women of reproductive age were our target because in most African households, it is typically the women’s responsibility to make sure the entire household is fed and women are more knowledgeable about the household food dynamics and dietary intake of all household members.

The questionnaire was designed to measure the food security status of the households by measuring household dietary diversity and food insecurity which reflects both household food availability and food access (48, 49). Information on household characteristics such as the household size, age, gender of household head, sources of food, income, land acquisition, wealth (assets acquired by household), and household monthly food expenditure were captured in the interviews. An index of wealth was created by combining information on the household’s possessions which included car/truck, motorbike, tractor, bicycle, fridge, television, radio, cattle/goats, chickens, cell phone, house, and electricity. For each household, the number of each asset was normalized (by dividing with the highest number obtained in each category for all households) then all summed to get a wealth index per household, which could range from 0 to 12 (47). Ethics approval was granted by the Rhodes University Ethical Standards Committee (permit number RU-HSD-14-08-0012).

### Household Dietary Diversity Scores (HDDS)

Household dietary diversity was determined using a standard 48-h recall technique (50). The respondents were to recall all foods and drinks consumed by the household members day and night in the previous 48 h during different meal times (before breakfast, breakfast, snacks before lunch, lunch, snacks before dinner, dinner, and snacks after dinner). The HDDS tool shows both household food availability and food access on the premise that households consume a variety of foods when they have the means to acquire them (48, 49). The tool inquires 17 food groups which are aggregated to 12 for analysis (49). The 12 food groups are: (1) cereals; (2) white tubers and roots; (3) vegetables; (4) fruits; (5) meat; (6) eggs; (7) fish and seafood; (8) legumes, nuts, and seeds; (9) milk and milk products; (10) oils and fats; (11) sweets; and (12) spices, condiments, and beverages. The score is the sum of food groups consumed by household members from the total of 12 (49, 51). Households were classified into classes of low-dietary diversity (less than or equal to five food groups), medium dietary diversity (six to seven food groups), and high-dietary diversity (greater than or equal to 8 food groups) (48), depending on the food groups they had consumed.

### Household Food Insecurity Access Scale (HFIAS)

The HFIAS is a continuous measure of the degree of food insecurity mostly related to access in the household in the past 30 days. We used the standardized HFIAS questionnaire composed of nine specific questions subdivided into three themes of food insecurity: (1) experiencing anxiety and uncertainty about the household food supply; (2) insufficient quality of diet including variety and preferences of the type of food; and (3) insufficient food intake or reducing quantity of food consumed (51). The questions address the situation of all household members and do not distinguish adults from children or men from women or adolescents.

The nine questions represent a generally increasing level of severity of food insecurity and nine “frequency-of-occurrence” questions were asked as a follow-up to each occurrence question to determine how often the condition occurred. A household was asked to describe how often a condition had occurred in the past 30 days if the response to the condition described in the corresponding occurrence question was yes. For each frequency-of-occurrence question, a score was assigned to each household: 1 if the response was rarely (condition having happened once or twice in the past 30 days); 2 if it occurred sometimes (3–10 times in the past 30 days) or 3 if the answer was often (occurred for more than 10 times in the past 30 days).

Households were assigned a score that ranged from 0 to 27 at the end of the nine questions which was based on their response to the nine questions (yes or no) and frequency-of-occurrence (rarely, sometimes, and often). Households were classified into different levels of food insecurity based on their response to the nine questions: 0 was assigned if the household responded “no” to all occurrence questions and a maximum score of 27 was given as a sum to a household whose response to all nine frequency-of-occurrence questions was “often” (51). A high-HFIAS score indicates household’s poor access to food and significant household food insecurity. Households were classified into classes of food secure (HFIAS = 0–1), mildly food insecure (HFIAS = 2–7), moderately food insecure (HFIAS = 8–11), and severely food insecure (HFIAS > 11) (51). A high-HFIAS score indicates household’s poor access to food and significant household food insecurity.

### Child Anthropometry

Nutritional status of children under the age of five is a good proxy indicator of a household’s nutrition status and health over a sustained period (52). Standing height and mid-upper arm circumference (MUAC) were measured (in triplicate using a calibrated rod and measuring tape to the nearest millimeter, respectively) on children (2–5 years) from interviewed households and where parents provided consent. The age (in months) and sex of the children were recorded. The children stood against the wall on a level floor without shoes, with legs, and heels placed together when stature was being measured. The measurement for MUAC was taken on the left upper arm, midway between the shoulder and elbow.

Standing height was used to calculate height-for-age (HAZ) *Z*-Scores (SD scores below or above a reference mean or median value). The WHO clear cut-off points were used to classify the children into classes of severe stunting (HAZ = <−3), moderate stunting (HAZ = −3 to <−2), mild stunting (HAZ = −2 to <−1), and normal (HAZ = ≥−1). These classes have different implications for food and nutrition situation of the household. For example, a child with a low HAZ (<−2) is stunted and is gaining insufficient height relative to their age, implying long-term malnutrition, and poor health (53, 54). Children were also classified into classes of wasting using the MUAC measurements according to WHO growth standards and guidelines (52, 55). The four classes were severe wasting (MUAC < 115 mm), moderate malnutrition (MUAC 115–125 mm), possibly mildly malnourished (MUAC 125–135 mm), and nutritionally normal (MUAC > 135 mm). An MUAC of <125 mm shows the child is wasted thereby implying acute malnutrition (54).

### Statistical Analysis

Data were entered and cleaned using Microsoft Excel and all statistical analyses were performed using Statistica version 12 (StatSoft Inc.). Descriptive data are presented as means and SDs and percentages. The differences in both HDDS and HFIAS between towns and locations were tested using two-way ANOVA with Bonferroni’s correction for multiple comparisons. As the data were not normally distributed, associations of the scores with food expenditure, household size, wealth variables, and access to use of land were examined through Spearman correlation tests and HDDS and HFIAS were used as response variables analyzed as a function of wealth, household size, food expenditure, and access to use of land. The association between HDDS and HFIAS was also tested using Spearman correlation. Statistical significance was set at *p* < 0.05.

## Results

### Dietary Diversity along the Agro-Ecological Gradient and Rural–Urban Continuum

The sample consisted of 554 women of reproductive age (15–49 years) with mean age of 32 ± 10 years. All household characteristics are given in Table 1.

**Table 1:** Comparison of household characteristics in study sites [adopted from Chakona and Shackleton (47)].

Variable	Richards Bay (*n* = 183)	Dundee (*n* = 173)	Harrismith (*n* = 198)	All (*n* = 554)
Respondent age (mean ± SD) (years)	29 ± 9.0	33 ± 10.8	33 ± 9.9	32 ± 10
Household size (mean ± SD) (number of people)	7 ± 4.6	8 ± 4.2	6 ± 2.2	7 ± 4
**Household head (%)**				
Male	42	36	47	42
Female	58	64	53	58
**Some form of cash income (%)**							
None	41	20	9	23
One income	49	59	72	60
Two or more incomes	10	21	19	17
Food expenditure (mean ± SD) (rand/week)	196 ± 180	333 ± 253	323 ± 271	284 ± 246
Wealth index	2.6 ± 0.6	2.3 ± 1.0	2.5 ± 0.9	2.5 ± 0.8
Households with land for own production (%)	73	57	27	52

**Figure 3:**
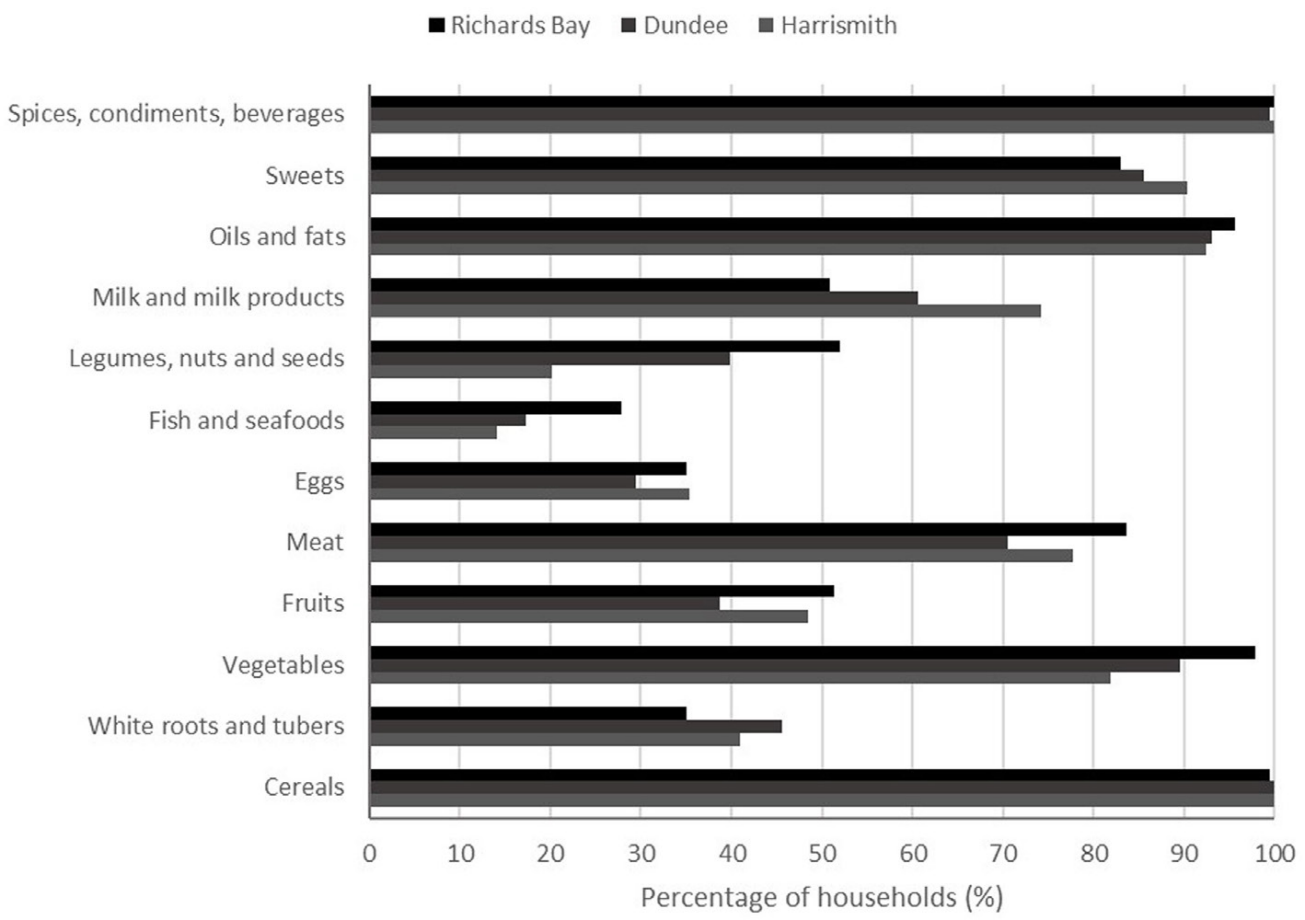
Percentage of households consuming different food groups in three towns of South Africa.

Diets were similar across the three sites (Figure 3). The food groups consumed by >50% of households were mostly cereals, vegetables (mostly cabbage and onion), spices, beverages, and condiments (mostly salt and curry powder), oils and fats; sweets (mostly sugar and sweetened juice), meat, and to a lesser extent milk and milk products (Figure 3).

Legumes, nuts and seeds, and fruits were consumed by more than 50% of households only in Richards Bay although fruits were frequently consumed in the other towns. Legumes were not commonly eaten in Dundee. White roots and tubers and eggs were hardly consumed in all the towns and households in Dundee consumed more tubers and fewer eggs than households in the other towns. Although Richards Bay had a greater percentage of households consuming fish and sea food, this food group was rarely consumed in all towns (Figure 3). In general, Richards Bay households had a better diet, being highest in oils, legumes, fish, eggs, meat, fruits, and vegetables.

The HDDS was generally low in all towns with a mean of 7.96 ± 1.60 for the sample (Table 2). Richards Bay had the highest mean HDDS of 8.44 ± 1.72 and Dundee had the lowest (7.69 ± 1.63). There was a significant difference in HDDS along the agro-ecological gradient (*F*_2, 545_ = 10.09, *p* < 0.001) being higher in Richards Bay than the other towns, which were not significantly different from one another (Table 2).

**Table 2:** Household Dietary Diversity Scores (HDDS) and the percentage of households with low, medium, and high HDDS in study sites (unlike superscripts indicate significant differences).

Town	HDDS (mean ± SD)	Location	HDDS (mean ± SD)	Percentage of households		
				Low	Medium	High
Richards Bay	8.44 ± 1.72^a^	Urban	8.43 ± 1.45^a^	2	23	75
		Peri-urban	8.26 ± 1.61^a^	5	46	49
		Rural	8.62 ± 1.33^a^	2	21	78

Dundee	7.76 ± 1.63^b^	Urban	8.18 ± 1.86^a^	8	16	76
		Peri-urban	7.57 ± 1.64^a^	9	35	56
		Rural	7.53 ± 1.55^a^	7	45	48

Harrismith	7.83 ± 1.59^b^	Urban	8.47 ± 1.26^a^	4	18	78
		Peri-urban	7.40 ± 1.64^b^	9	42	48
		Rural	7.60 ± 1.61^b^	10	55	34

All	7.96 ± 1.60	Urban	8.38 ± 1.50^a^	6	31	63
		Peri-urban	7.73 ± 1.67^b^			
		Rural	7.93 ± 1.53^b^			

Within sites, mean HDDS ranged from 7.53 ± 1.55 to 8.62 ± 1.33 in the rural location, 7.40 ± 1.64 to 8.26 ± 1.61 in the peri-urban location, and 8.18 ± 1.86 to 8.47 ± 1.26 in the urban location (Table 2). There was a significant difference along the rural–urban continuum (*F*_2, 545_ = 6.90, *p* < 0.001) being higher in the urban than the other two, which were not significantly different. Significant differences were observed along the rural–urban continuum in Harrismith (Table 2). In Richards Bay and Harrismith, HDDS were low in the peri-urban locations and high in the urban location in Harrismith and rural in Richards Bay. This was different to Dundee where HDDS were low in rural locations and high in the urban. Most households in all towns and locations were classified in the high-HDDS group except for rural Harrismith where the majority (55%) had medium HDDS (Table 2).

### Household Food Access along the Agro-Ecological Gradient and Rural–Urban Continuum

The HFIAS was generally low in all towns with a mean of 7.07 ± 7.06 for the sample (Table 3). About 36% of the households were food secure, 24% were mildly food insecure, 28% were moderately food insecure, and 12% were severely food insecure (Table 3). The level of household food access was high in Richards Bay with the lowest HFIAS score of 5.57 ± 6.98 and food access was low in Dundee which had the highest HFIAS of 9.39 ± 7.13 (Table 3). There was a significant difference in HFIAS along the agro-ecological gradient (*F*_2, 545_ = 12.13, *p* < 0.001) with Dundee being more food insecure than the other two, which were not significantly different to one another. Within sites, mean HFIAS ranged from 6.74 ± 6.21 to 9.45 ± 6.67 in the rural location, 5.49 ± 6.89 to 11.37 ± 7.19 in the peri-urban location, and 3.02 ± 6.29 to 5.37 ± 6.49 in the urban location (Table 3). The lowest mean HFIAS was recorded in Richards Bay urban and the highest was recorded in Dundee’s peri-urban location (Table 3). There was a significant difference in HFIAS along the rural–urban continuum (*F*_2, 545_ = 21.80, *p* < 0.001) with urban households having lower scores than the other two locations, which were not significantly different (Table 3). This was true for all sites. The prevalence of household food insecurity was much higher in peri-urban areas for Dundee and Harrismith and Richards Bay rural (Table 3). The urban population in all three towns was more food secure than the rural and peri-urban populations. However, Dundee had the greatest prevalence of severe food insecurity in the peri-urban and lowest in urban location (Table 3).

**Table 3:** Percentage of households who are food insecure and food secure classified using Household Food Insecurity Access Scale (HFIAS) (unlike superscripts indicate significant differences).

Town	HFIAS (mean ± SD)	Location	HFIAS (mean ± SD)	Percentage of households			
				Severely food insecure	Moderately food insecure	Mildly food insecure	Food secure
Richards Bay	5.57 ± 6.98^b^	Urban	3.02 ± 6.29^a^	9	2	23	66
		Peri-urban	5.49 ± 6.89^a/b^	8	22	20	50
		Rural	7.44 ± 7.07^b^	14	19	35	32

Dundee	9.39 ± 7.13^a^	Urban	5.37 ± 6.45^a^	3	32	13	53
		Peri-urban	11.37 ± 7.19^b^	27	41	19	13
		Rural	9.45 ± 6.67^b^	15	42	30	13

Harrismith	6.43 ± 6.59^b^	Urban	3.18 ± 6.02^a^	4	16	7	73
		Peri-urban	8.32 ± 6.46^b^	13	36	29	21
		Rural	6.74 ± 6.21^b^	7	31	31	31

Mean	7.07 ± 7.06	Urban	3.74 ± 6.27^a^	12	28	24	36
		Peri-urban	8.38 ± 7.21^b^				
		Rural	7.88 ± 6.67^b^				

### Association between Food Access Indicators and Socio-Economic Indicators

Significant correlations were observed between HDDS and HFIAS, and household attributes (Table 4).

**Table 4:** Spearman correlations between Household Dietary Diversity Scores (HDDS) and Household Food Insecurity Access Scale (HFIAS) and selected socio-economic indicators in the three towns studied (correlations are significant at *p* < 0.001^a^, *p* < 0.01^b^, and *p* < 0.05^c^).

Town	HDDS vs. HFIAS		Household size	Food expenditure	Wealth index	Access to land
Richards Bay	−0.286^c^	HDDS	0.054	0.101	0.132	−0.038
		HFIAS	0.068	−0.058	−0.096	0.141

Dundee	−0.342^b^	HDDS	−0.077	0.337^c^	0.278^c^	0.079
		HFIAS	0.195^c^	−0.354^b^	−0.337^b^	−0.057

Harrismith	−0.474^a^	HDDS	−0.023	0.185^b^	0.081	0.043
		HFIAS	−0.052	−0.255^a^	−0.145^a^	−0.079

All	−0.464^b^	HDDS	−0.058	0.066	0.109^b^	0.132^a^
		HFIAS	0.050	−0.073	−0.105^c^	−0.038

There was a significant negative correlation between HDDS and HFIAS in all towns. In Dundee, both HFIAS (negative) and HDDS (positive) were correlated with household wealth index, food expenditure as well as a significant correlation between HFIAS and household size (Table 4). In Harrismith, the only significant correlations were between HDDS and food expenditure and between HFIAS and wealth and food expenditure. No significant relationships between HDDS/HFIAS and household characteristics were observed in Richards Bay.

### Nutritional Status of Children along the Agro-Ecological Gradient

The sample consisted of 216 children; 69 from Richards Bay, 83 from Dundee, and 64 from Harrismith. Of these, 21 were from urban areas, 104 from peri-urban, and 91 from the rural areas. The mean age of children was 39.0 ± 11.2 months and 53% were females. The mean HAZ for the sample was −0.93 ± 4.94 and mean MUAC was 152.1 ± 24.9 mm. Richards Bay had the greatest percentage of children who were stunted and the least who were wasted (Table 5). Harrismith had the greatest percentage of children who were severely stunted and who were severely wasted (Table 5). There were no significant differences in stunting along the agro-ecological gradient, along the rural–urban continuum or between sex of child. Significant differences were observed in child wasting along the agro-ecological gradient (*F*_2, 210_ = 6.65, *p* = 0.002) with Harrismith having more wasted children than the other towns, which were not significantly different from each other. Male children had higher MUAC than female (*F*_1, 210_ = 4.06, *p* < 0.05). There were no significant differences in MUAC along the rural–urban continuum.

**Table 5:** Anthropometric measurements of children 2–5 years old and percentages of stunted and wasted children using height-for-age (HAZ) and mid-upper arm circumference, respectively (unlike superscripts indicate significant differences).

	Town/location	Mean ± SD	Severe stunting/wasting	Moderate stunting/wasting	Mild stunting/wasting	Normal
HAZ	Richards Bay	−1.65 ± 1.88^a^	23	22	19	36
	Dundee	−0.63 ± 1.83^a^	10	13	16	61
	Harrismith	−1.54 ± 2.18^a^	25	14	13	48
	Urban	−1.89 ± 1.32^a^	24	19	10	48
	Peri-urban	−0.82 ± 1.22^a^	16	18	20	45
	Rural	−0.68 ± 1.28^a^	20	13	13	54
	All	−0.93 ± 4.94	19	16	16	48

MUA C	Richards Bay	159.4 ± 22.5^a^	1	9	16	74
	Dundee	151.6 ± 24.8^a^	7	11	16	66
	Harrismith	145.0 ± 25.5^b^	13	13	20	55
	Urban	149.8 ± 25.9^a^	10	10	19	62
	Peri-urban	154.2 ± 26.1^a^	5	15	13	66
	Rural	150.3 ± 23.2^a^	9	5	21	65
	All	152.1 ± 24.9	7	11	17	65

The prevalence of wasting followed the agro-ecological gradient with the lowest percentage in Richards Bay and the highest in Harrismith. Child stunting was highest in Richards Bay and lowest in Dundee. Stunting was significantly associated with food access as measured by HFIAS for the sample (*r_s_* = 0.16, *p* < 0.02). Stunting was also significantly associated with wasting in Richards Bay with severely stunted children being severely wasted (*r_s_* = 0.37, *p* < 0.001). Wasting in Harrismith was significantly negatively associated with HDDS (*r_s_* = 0.27, *p* < 0.05), with children from households with low HDDS having large MUAC. There were no significant correlations between HFIAS and wasting in all towns. The prevalence of wasting and stunting followed the rural–urban continuum with the lowest percentages of both indicators in the rural areas and highest in the urban areas. When the urban sample was removed from the analysis due to low-sample size, the prevalence of both stunting and wasting was higher in the peri-urban than the rural locations.

## Discussion

### Household Food Insecurity along the Agro-Ecological Gradient as Measured by HDDS and HFIAS

The use of dietary diversity as an indicator of dietary quality is widespread in food security studies at both individual and household levels. Households in all three towns had a poor dietary diversity with one in three households having diets lacking good quality and diverse foods rich in all essential nutrients, as has been reported in many South African studies (31, 56). About 64% of the households were food insecure, with 12% being severely food insecure. The fewer food groups a household consumed, the higher the level of food insecurity. The most affected were the peri-urban and rural households. The percentage of the food secure households (36%) in this study is lower than the 46% reported by Shisana et al. (38) and 43% reported by Rose and Charlton (57). As was hypothesized, the prevalence of food insecurity was highest in peri-urban and rural locations following Shisana et al. (38) although Rose and Charlton (57) found the greatest percentages of food insecurity in rural households.

In this study, a large percentage of households restricted their food consumption to a number of food groups which is insensitive to geographical location and poverty status of households. Greater than 50% of the households across all towns and locations consumed similar food groups [cereals (mostly maize meal), oil/fat, vegetables (mostly cabbage and onion), meat, sweets, and spices]. This is consistent with Rabbani (18) who found similar trends in Bangladesh. The consumption of other food groups was limited to town and location, as explained by the significant differences in HDDS evident along the agro-ecological gradient and rural–urban continuum. Households in Richards Bay had high levels of food access and dietary diversity which could be attributed to the better agro-ecological potential which favors agriculture and reduces dependence on food purchasing. A greater percentage of households in this town consumed diverse foods unlike households in Dundee and Harrismith who were not producing their own food and were net buyers, hence had limited access to food which increased their level of food insecurity. Dietary diversity was strongly correlated with access to use of land in this study, reflecting that households with land have the greater engagement in own production which improves the quality of diets. Farming has been shown to increase food security for low-income households as farm produce become an alternative to imported foodstuffs which is cost effective for poor households as this may lower dependence on food purchasing. However, in South Africa, decline in agriculture and reliance on purchased food is negatively impacting households’ food security (58).

Significant positive correlations observed between HDDS and food expenditure in Dundee and Harrismith, and between HDDS and wealth in Dundee explain the association of dietary diversity and household socio-economic status. This is consistent with Hatløy et al. (59) in Mali and Hoddinott and Yohannes (60) and Savy et al. (61) in Burkina Faso as some food groups may be limited to higher income households (18). This was not true for Richards Bay as households had greater access to a variety of foods especially legumes, wild vegetables, fruits, and fish which were less consumed in the other towns. Furthermore, household size has been shown to significantly affect household food access, especially in Dundee. Thus, levels of households’ food security may vary between and within towns and locations due to other stressors hindering the ability of a household to access food and in this case household size. Therefore, households in the same geographic location may face different levels of food insecurity due to other factors like the economic crisis and stressors such as illness or unexpected expenses (35, 62).

In all towns, levels of food insecurity were lower in urban areas compared with rural and peri-urban areas with the peri-urban households being the most affected. In Dundee alone, about 27% of the households were severely food insecure in peri-urban zone. On average, households in urban localities consumed more food groups; followed by rural and lastly the peri-urban households and was opposite for HFIAS with the peri-urban having the highest scores. These results indicate that populations in the peri-urban are the most vulnerable to food insecurity as was hypothesized in this study. This supports other studies (62–64) noting that food insecurity affects the urban poor (mostly informal settlements) more severely than their rural counterparts. In urban areas, food is readily available but poor households have limited access (especially the peri-urban dwellers), due to high levels of poverty, unemployment, and limited access to land. In these areas, household income and wealth determine the level of household food security as by strong correlations between wealth, food expenditure, and HDDS as well as HFIAS. In this case, low-income households may experience food shortages more than wealthier households as food expenditure makes up a large share of their cash spending therefore they are more vulnerable to the impacts of high-food prices. This study supports reports by Oldewage-Theron et al. (56) and Labadarios et al. (31) in South Africa that poor socio-economic status has an impact on household food insecurity in peri-urban locations. Therefore, poor households cope with poverty and rising food prices by reducing the quality, quantity, and number of meals consumed per day. This is supported by this study as households with greater access to food consume more food groups and vice-versa as was also reported by FAO (48). Deitchler et al. (65), Heady and Ecker (66), Sahyoun et al. (67), and Desiere et al. (68) also argued that HFIAS is correlated with food intake-based measures of food security and Hatløy et al. (59) and Hoddinott and Yohannes (60) noted that an increase in dietary diversity is associated with higher socio-economic status and household food security. Therefore, the study also identified that food insecurity in South Africa is closely linked to poverty.

### Household Food Insecurity and Child Malnutrition

Stunted growth was the dominant form of malnutrition among children in the study sites with 35% of the children suffering from chronic malnutrition. This percentage is higher than 27% that was reported by Shisana et al. (38) for South African children under the age of three although it is within the range of 21–48% from other studies (22, 36, 37). The percentage of children who are wasted (18%) was far >4.5% that was reported for South Africa in 2005 (39) and 6.8% (4.8% wasted and 2% severely wasted) in 2008 (40). The percentages of children who were suffering from wasting was alarming, with Harrismith having the highest prevalence. This is consistent with Faber and Wenhold (41) who reported that chronic malnutrition is a bigger problem than acute malnutrition in South Africa and Saaka and Osman (9) also reported a high prevalence of chronic malnutrition in Ghana. Fruits and vegetables (Vitamin A rich and dark green leafy) were infrequently consumed in this study, which is consistent with Labadarios et al. (43) who reported that poor access and availability restricts South African children from consuming adequate fruits and vegetables. Steyn et al. (16) reported that low-dietary diversity was associated with poor growth in South African children. In a study across 11 countries, Arimond and Ruel (5) confirmed that child nutritional status is associated with household dietary diversity. The present study further indicated that stunting and wasting were significantly positively correlated in Richards Bay where stunted children were more likely to be wasted.

Food insecurity is related to an inadequate dietary intake and increased levels of stunting and underweight (43). This study has shown that low-dietary diversity (HDDS) is negatively associated with wasting in Harrismith where children from households with low HDDS had large MUAC which could be attributed to high consumption of poor quality diets rich in more energy foods, more processed foods, and foods high in saturated fat, sugar, and salt which causes overweight (7, 28, 69). Changes in diet have been reported to be one of the main drivers to child overweight (7, 69). Also, the study has shown that household food access is positively correlated to stunting, as children from households with poor food access were mostly likely to be stunted. The study has shown that poor households are more likely to suffer from poor food access and poor dietary diversity which in turn affects the nutritional status of young children as these households may have wasted and/or stunted children as well as obese children which is affecting the affecting the majority of the population in South Africa (28, 31). Early childhood malnutrition has been linked to poverty and lack of economic resources (7). This study concludes that children’s nutritional status is related to household food security, socio-economic status, and local context. Any limitations in household income and access to food can result in different forms of nutritional status in children as households may decrease dietary quality and quantities to cope with food shortages (56, 70). The significant negative correlation between HDDS and wasting in Harrismith suggests that poverty could be the major determining factor of the nutritional status of children in this town as poor households may resort to inadequate meals of cheap and poor quality (more energy and more processed foods, including refined grains, and foods high in saturated fat, sugar, and salt) to cope with poverty and rising food prices as many are net buyers of food and not practising subsistence farming. Large MUAC of children in Harrismith could be due to high intake of dietary fat and low consumption of fruits and vegetables as was the case of Hispanic children in the USA (71). Labadarios et al. (31) also indicated that poor South African children consumed inadequate diets which did not meet nutritional requirements indicated by the limited variety of foods they consumed. However, the nutritional status of children in this study depend on the choice and consumption of diverse foods among these households which is determined by a household’s ability to access food and socio-economic status.

Nutritional status of children in Dundee was not associated with levels of food insecurity, despite higher levels of food insecurity observed in the town. Factors other than dietary diversity and food access could be attributed to this observation as food security alone may not ensure nutritional security. Intra-household allocation of food should not be ignored. The nutritional status of young children in Dundee could have been less sensitive to household food insecurity as households may reallocate resources when food is scarce to buffer the youngest children from declines in food intakes (9). Thus, poor households would cope with food insecurity by reducing the quality, quantity, and number of meals consumed per day, without sacrificing nutrient adequacy for vulnerable members (9), in this case small children. For example, some mothers may prioritize their children’s food consumption over their own (72). Households who are engaging in farming and are producing a major share of their food are less likely to suffer from food insecurity than households which depend almost entirely on purchased food (47). Children from these households are less likely to be malnourished, which is evident in this study as the prevalence of wasting followed the agro-ecological gradient with Richards Bay having the lowest and Harrismith the highest. However, stunting was higher in Richards Bay than the other towns. In this case, other factors such as recurrent infections, inadequate sanitation and hygiene, maternal education, and less than optimal infant and young child feeding practices (13, 73), consuming low-nutrient density foods (74) as well as maternal and antenatal factors (13) could have contributed to stunting in Richards Bay. Access to food as measured by HFIAS was associated with stunting, which is consistent with Hadley et al. (12) although some studies (4, 9, 75) have not shown significant associations between stunting in children and food insecurity. This study also revealed that the prevalence of food insecurity and child malnutrition was higher in peri-urban locations which is consistent with Berry et al. (40).

Agro-ecological potential has significant influence on children’s nutritional status, which is also related to household food security and socio-economic status. Dependence on food purchasing and any limitations in households’ income, access to land and food, can result in different forms of nutritional status in small children. Considering that there were no significant differences in stunting along the agro-ecological gradient, along the rural–urban continuum, and between sex of the child, it is a clear indication that responses to address stunting in South Africa need to be prioritized and move beyond relying on food security interventions, including grants, backyard or community gardens, and nutritional-specific interventions. Indeed, a coherent response that effectively brings nutrition-specific programs (primary and maternal health, water and sanitation, micronutrient supplementation or fortification, exclusive breastfeeding and complementary feeding, dietary supplementation for children, dietary diversification, and nutrition interventions in emergencies), nutrition sensitive programs and approaches (agriculture and food security especially food access, gender, social safety nets, e.g., child support grants, school feeding programs, early child development, women’s empowerment, classroom education, water, and sanitation); and building an enabling environment (rigorous evaluations of food security and nutrition programs, advocacy strategies, accountability, incentives regulation, and legislation as well as domestic resource mobilization) (7), together remains a major challenge which need to be addressed. Policy makers in South Africa need to include the study sites as emergence areas for nutrition interventions and also do rigorous evaluations and accountability on the existing nutrition sensitive programs in the country such as the child support grant and school feeding programs. Furthermore, there is need for promoting the practise of subsistence agriculture (including climate-smart agriculture especially in drier areas) and consumption of wild foods as these have been noted to be some of the determinants of food security and nutrition. The association between access to land and dietary diversity along the agro-ecological gradient has an important implication for municipalities to make the land available to affected households so that they diversify food access through production of their own food and lower dependence on food purchasing.

## Ethics Statement

This study was carried out in accordance with the recommendations of Rhodes University ethics guidelines, Rhodes University Ethical Standards Committee. The protocol was approved by the Rhodes University Ethical Standards Committee.

## Author Contributions

Substantial contributions to the conception or design of the work (GC and CS); or the acquisition, analysis, or interpretation of data for the work (GC); AND Drafting the work (GC) or revising it critically for important intellectual content (GC and CS); AND Final approval of the version to be published (GC and CS); AND Agreement to be accountable for all aspects of the work in ensuring that questions related to the accuracy or integrity of any part of the work are appropriately investigated and resolved (GC and CS).

## Conflict of Interest Statement

The authors declare that the research was conducted in the absence of any commercial or financial relationships that could be construed as a potential conflict of interest.
